# X-Linked Alport Syndrome in Women: Genotype and Clinical Course in 24 Cases

**DOI:** 10.3389/fmed.2020.580376

**Published:** 2020-11-23

**Authors:** Antonio Mastrangelo, Marisa Giani, Elena Groppali, Pierangela Castorina, Giulia Soldà, Michela Robusto, Chiara Fallerini, Mirella Bruttini, Alessandra Renieri, Giovanni Montini

**Affiliations:** ^1^Pediatric Nephrology, Dialysis and Transplant Unit, Fondazione IRCCS Ca' Granda Ospedale Maggiore Policlinico, Milan, Italy; ^2^Department of Pediatrics, V. Buzzi Children's Hospital, Milan, Italy; ^3^Casa di Cura Igea, Milan, Italy; ^4^Dipartimento di Scienze Biomediche, Humanitas University, Milan, Italy; ^5^Humanitas Clinical and Research Center, Milan, Italy; ^6^Experimental Therapeutics Program, Istituto FIRC di Oncologia Molecolare-Fondazione Italiana per la Ricerca sul Cancro Institute of Molecular Oncology Foundation, Milan, Italy; ^7^Medical Genetics, University of Siena, Siena, Italy; ^8^Azienda Ospedaliera Universitaria Senese, Medical Genetics, University of Siena, Siena, Italy; ^9^Giuliana and Bernardo Caprotti Chair of Pediatrics, Department of Clinical Sciences and Community Health, University of Milan, Milan, Italy

**Keywords:** Alport syndrome (AS), genotype phenotype correlation, female, X-linked, proteinuria

## Abstract

**Objectives:** X-linked Alport syndrome (XLAS) females are at risk of developing proteinuria and chronic kidney damage (CKD). The aim of this study is to evaluate the genotype-phenotype correlation in this rare population.

**Materials and Methods:** This is a prospective, observational study of XLAS females, confirmed by a pathogenic mutation in *COL4A5* and renal ultrastructural evaluation. Proteinuria, renal function and extrarenal involvement were monitored during follow-up. Patients were divided in 2 groups, according to mutations in *COL4A5*: missense (Group 1) and non-missense variants (Group 2).

**Results:** Twenty-four XLAS females, aged 10.6 ± 10.4 years at clinical onset (mean follow-up: 13.1 ± 12.6 years) were recruited between 2000 and 2017 at a single center. In group 1 there were 10 patients and in group 2, 14 (mean age at the end of follow-up: 24.9 ± 13.6 and 23.2 ± 13.8 years, respectively). One patient in Group 1 and 9 in Group 2 (*p* = 0.013) developed proteinuria during follow-up. Mean eGFR at last follow-up was lower in Group 2 (*p* = 0.027), where two patients developed CKD. No differences in hearing loss were documented among the two groups. Two patients in Group 2 carried one mutation in both *COL4A5* and *COL4A3* (digenic inheritance) and were proteinuric. In one family, the mother presented only hematuria while the daughter was proteinuric and presented a greater inactivation of the X chromosome carrying the wild-type allele.

**Conclusions:** The appearance of proteinuria and CKD is more frequent in patients with severe variants. Carrying digenic inheritance and skewed XCI seem to be additional risk factors for proteinuria in XLAS females.

## Introduction

Alport syndrome (AS) is a rare but progressive, inherited nephropathy characterized by microscopic hematuria, proteinuria, progressive renal failure and ultrastructural lesions of the glomerular basement membrane (GBM). It is often associated with sensorineural deafness and ocular abnormalities and is caused by mutations in the *COL4A3, COL4A4*, and *COL4A5* genes, which encode collagen IV chains.

The first two genes, *COL4A3* and *COL4A4*, are located on chromosome 2, while *COL4A5* is located on chromosome X. Inheritance can be X-linked (65–80%), autosomal recessive (15%) or autosomal dominant (about 5–20% of families). Nearly 90% of males with X-linked Alport syndrome (XLAS) develop early chronic kidney disease (CKD) and then end-stage kidney disease (ESKD) by the age of 40 years. As it was thought that females had a benign renal course, they were initially described as “benign carriers”: in 1927 Alport himself reported that “the females have deafness and hematuria and live to old age” (1). However, it has subsequently been shown that women are also at risk for developing ESKD and that the risk of progressive renal failure in females increases with age: about 15–30% reach ESKD by 60 years of age, and often suffer hearing loss in middle age (2). Moreover, an affected female will transmit the mutation to half of her sons and half of her daughters. For all these reasons, the diagnosis of XLAS in women is mandatory.

In their meta-analysis of genotype-phenotype correlation in XLAS males, Gross et al. found that the type of mutation is a significant predictor of disease severity (3). Furthermore, they showed that glycine substitutions in exon 1–20 resulted in a milder phenotype. Bekheirnia et al. discovered that the average age at onset of ESKD in XLAS males was 37 years for those with missense mutations, 28 years for those with splice-site mutations, and 25 years for those with truncating mutations (*p* < 0.0001) (4).

While the genotype-phenotype correlation in men is clear, it remains poorly characterized in women. Few studies have investigated the genotype-phenotype correlation in females, most of these being case-reports, with contradictory results (2). Jais et al. reported the lowest rate of progression to ESKD in female patients with missense mutations, but this finding was not statistically significant (5). They also found that the appearance of proteinuria represented a negative prognostic criterion, as well as deafness.

In this regard, the international Alport mutation consortium stigmatizes the importance of random X-chromosome inactivation (XCI) in females (6). Additionally, the presence of a second mutation (digenic inheritance) may play a role in some cases, so the correlation can be very challenging (7).

Savige et al. studied genotype-phenotype correlation in 68 adult females: 39% of the so-called severe mutations were seen in 23 patients developing ESKD vs. 20% in 45 without ESKD; even in this case, no statistical significance was obtained (8). In this study, severe mutations included copy number variants, non-sense, splice site, and frameshift mutations located both in the collagenous domain and the NC1 domain. Yamamura et al. recently demonstrated a lack of genotype-phenotype correlation in a very large cohort of Japanese females with XLAS (9). However, differences in ethnicity and patient recruitment could have led to different results.

The aim of our study is to better define the genotype-phenotype correlation in this rare population.

## Materials and Methods

### Patients

We conducted a prospective, observational analysis on female patients with XLAS. Eligible patients and their families were recruited from the outpatient clinics of a single center, the Pediatric Nephrology, Dialysis and Transplant Unit, in Milan between 2000 and 2017. All patients were clinically evaluated by the same renal physician. A diagnosis of XLAS was made in females carrying the characteristic clinical features of the disease, after a pathogenic mutation in the *COL4A5* gene was shown by genetic analysis. Similarly to what was defined by Savige et al. (8), we divided our patients in two groups according to mutations in COL4A5: Group 1 included patients with missense mutations (Mild forms) and in Group 2 were the patients with any type of non-missense variants (Severe forms) and those with complex genotypes, such as those with digenic mutations (see Genetic Analysis section).

### Inclusion Criteria

Inclusion criteria were:

- female gender
- presence of persistent glomerular hematuria
- genetic analysis showing a pathogenic mutation in *COL4A5* gene (see Genetic Analysis section)

associated with at least one of the following:

- renal biopsy suggestive for AS (Electron microscopy showing lamellated GBM and/or thinning and thickening of GBM and/or basket weave lesions and/or associated glomerular sclerosis)
- familiar history positive for AS or for persistent glomerular hematuria
- sensory-neural hearing loss

Exclusion criteria:

- absence of microscopic hematuria tested every 3 months on 4 consecutive occasions
- presence of other superimposed glomerular diseases and/or dysplastic kidneys and/or associated systemic disease

### Clinical Data

Clinical data regarding family status, age at diagnosis, presence of proteinuria, estimated Glomerular Filtration Rate (eGFR), hearing loss and ocular lesions were evaluated at onset and monitored during follow-up.

Proteinuria was quantified by spot urinary Protein-to-Creatinine ratio (uPr/uCr) expressed in mg/mg (10) and considered pathologic if >0.2 mg/mg in 3 consecutive samples during a 6 month observation period. During the clinical follow-up period the uPr/uCr was evaluated every 3 months in all XLAS patients, in addition to annual audiometric and ophthalmologic examinations. Angiotensin Converting Enzyme inhibition (ACE-I) was initiated when uPr/uCr was ≥ 0.5 mg/mg.

Blood pressure was monitored during the entire follow up period. We defined hypertension as arterial blood pressure above the 95th percentile for age and sex.

Estimated GFRs were calculated using the Schwartz formula (k 0.55) for patients <18 years old (11) and the MDRD formula for those ≥18 years old (12).

### Genetic Analysis

Mutation screening of *COL4A3/COL4A4/COL4A5* was performed by locus-specific amplification followed by massively parallel sequencing (454 Junior sequencing Roche, Basel, Switzerland). The pathogenetic variants identified in probands were confirmed by direct Sanger sequencing in 22/24 females. Patient n°1 in Group 1 and patient n°8 in Group 2 were analyzed by Denaturing High Performance Liquid Chromatography (DHPLC) only and did not consent to subsequent investigations. All variants were checked in the following online databases: NCBI dbSNP Build 153 https://www.ncbi.nlm.nih.gov/projects/SNP/snp_summary.cgi, ExAC (Exome Aggregation Consortium; http://exac.broadinstitute.org/) and 1000 genomes (http://www.1000genomes.org). We also consulted published manuscripts on the subject, the *COL4A5* LOVD database (https://databases.lovd.nl/shared/genes/COL4A5) and the ARUP *COL4A5* mutation database (https://arup.utah.edu/database/ALPORT/ALPORT_welcome.php; Last update: December 2017) to check whether variants had already been reported in AS. For splicing variants, we performed *in silico* evaluation of pathogenicity using Alamut software v2.11 (Interactive Biosoftware, Rouen, France), which includes the following splicing prediction tools: SpliceSiteFinder-like, MaxEntScan, NNSPLICE, GeneSplicer. Finally, for missense variants we checked the pathogenicity using the "Combined Annotation Dependent Depletion tool (CADD, https://cadd.gs.washington.edu/).

Regarding the prioritization of the variants, we considered the localization, or not, in the collagen domain of the missense variants involving a glycine, the effect on the protein (protein reconstruction software?), the result of the prediction tools (CADD / Alamut).

Skewing of XCI in two female carriers of the *COL4A5* c.2245-40A>G mutation was investigated by analyzing a polymorphic trinucleotide repeat in the androgen receptor gene, for which the tested females were heterozygous, by means of a methylation-sensitive restriction enzyme assay, as previously described (13).

According to previous studies reported in the literature (5, 8), we defined two types of mutation: missense (Mild forms) and non-missense variants (Severe forms). In the latter group, we considered all non-missense mutations located both in the collagenous and NC1 domains: large deletions, rearrangements, premature stop codons, frameshift mutations, and mutations involving the splice site. We also included patients with a complex phenotype, such as digenic mutations.

Informed consent for clinical data and DNA analysis was obtained from each patient or parents/Legally Authorized Representative for minors, before enrolment.

### Ultrastructural Analysis

Specimens derived from renal biopsy underwent ultrastructural analysis by electron microscopy (EM). The EM picture was considered suggestive for AS in presence of lamellated GBM and/or thinning and thickening of GBM and/or basket weave lesions and/or associated glomerular sclerosis, while thin basement membrane disease (TBMD) was characterized by the presence of diffuse thinning of GBM in the absence of other features.

### Statistical Analysis

Statistical analysis was performed using Graphpad prism software version 8.1.2. Continuous variables were summarized as mean ± standard deviation.

The Mann-Whitney U test was used to compare the distribution of continuous variables in groups. The association between categorical variables was evaluated using Fisher's exact test.

## Results

Twenty-four females with XLAS were enrolled in the study. Mean age was 10.7 ± 10.4 years at clinical diagnosis, and 21.2 ± 13.9 years at genetic diagnosis. Mean follow-up was 13.1 ± 12.6 years. All patients but one (Chinese) were of Caucasian ethnicity. Group 1 consisted of 10 patients (mean follow up 12 ± 12.7 years, age at last follow up 24.9 ± 13.6 years) and Group 2 of 14 patients (mean follow up 14 ± 13.1 years, age at last follow up 23.2 ± 13.8 years). Both mean follow up and age at last follow up visit were very similar between the two groups (*p* = 0.541 and *p* = 0.659, respectively).

The complete clinical characteristics of the patients are shown in Supplementary Table 1 and summarized in Table 1. In our cohort, hematuria was observed in all patients, by definition, while proteinuria was detected in 10/24 (41%).

**Table 1:** Clinical features of Group 1 and Group 2.

	**Age at last follow up visit Mean ± SD**	**Number of patients with proteinuria**	**Age at onset of uPr/uCr >0.5 mg/mg (years) Mean ± SD**	**eGFR at last follow-up (ml/min/1.73 sm)**	**Number of patients with ESKD or CKD**	**Number of patients with Deafness**	**Number of patients with Ocular abnormalities**
Group 1 (no. 10)	24.9 ± 13.6 years	1/10	9	119.5 ± 6.3	0/10	2/10	0/10
Group 2 (no. 14)	23.2 ± 13.8 years	9/14	15.1 ± 9.2	103.4 ± 30.0	2/14	2/14	0/14
p	n.s.	*p* = 0.013	n.s	*p* = 0.027	n.s.	n.s.	n.s.

*n.s., not significant*.

### Genetic Analysis

The vast majority of the patients (21/24) had a family history positive for AS. A pathogenetic mutation was detected in all affected members. The genetic data from our patients are summarized in Table 2. Three patients carried *de novo* mutations without a family history of XLAS; one patient was an adopted child and their family history was not available. According to the classification proposed by Savige et al. (8), Mild mutations in Group 1 were Gly-XY missense in 9 patients and Cys-Tyr missense in one patient. Among these patients, only one was proteinuric, with a Gly-Arg mutation. On the other hand, in Group 2, 2 patients presented with splicing acceptor mutations, one with a mutation involving the donor splice site, one with an inframe mutation, 3 with non-sense mutations, 5 with frameshift, one of whom (n°12) also had a mutation in the *COL4A3* gene (missense); another patient (n°13) had a digenic mutation (*COL4A5* missense and *COL4A3* deletion). Patient n°13 developed SNHL and progressed to ESKD. Finally, in patient n°14, a novel heterozygous intronic variant (c.2245-40A>G), potentially disrupting COL4A5 exon29 splicing, was identified. She developed proteinuria at 13 years of age. Using a minigene-based approach in HEK293 cells, we previously demonstrated that this variant abolishes exon29 branch site (13). The variant was also present in her mother (Patient n°2), who showed only microscopic hematuria. This difference in the phenotype between mother and daughter correlates with the levels of inactivation of the X-chromosome carrying the wild type allele, as assessed by a methylation-sensitive restriction-enzyme assay (13). Indeed, the wild-type c.2245-40A allele showed a very high level of inactivation (91%) in the proteinuric daughter (Patient n°14), whereas the non-proteinuric mother (Patient n°2) had a balanced inactivation of both alleles (45% vs. 55%).

**Table 2:** Mutations identified in our cohort.

**Pts**	**Nucleotide position**	**Mutation type and aminoacidic position**	**Exon/Intron**	**dbSNP; References**	**CADD^§^/Alamut**	**Pathogenicity**
**GROUP 1**—**patients with Mild mutations**						
1	c.1700G>C	Missense p. (Gly567Ala)	24	rs104886137 (14)	26^§^	R
2	c.2473G>A	Missense p. (Gly825Arg)	30	None	27^§^	P
3	c.1957G>T	Missense p. (Gly653Trp)	26	LOVD database	38^§^	R
4	c.1835G>A	Missense p. (Gly612Asp)	25	rs281874680 (15)	28^§^	R
5	c.3695G>C	Missense p. (Gly1232Ala)	41	LOVD database	27^§^	R
6	c.4562G>A	Missense p. (Cys1521Tyr)	48	LOVD database	31^§^	R
7	c.938G>T	Missense p. (Gly313Val)	17	LOVD database	33^§^	R
8	c.629G>A	Missense p. (Gly210Glu)	11	LOVD database	25^§^	R
9	c.2918G>A	Missense p. (Gly973Asp)	34	(16)	31^§^	R
10	c.2077G>A	Missense p. (Gly693Arg)	27	(17)	28^§^	R
**Pts**	**Nucleotide position**	**Mutation type and aminoacidic position**	**Exon/Intron**	**dbSNP; References**	**CADD^§^/Alamut^†^**	**Pathogenicity**
**GROUP 2**—**patients with Severe mutations**						
1	c.546+5G>C	Acceptor splice site	Intron 9	None	Affects splicing	P
2	c.2245-40A>G^*^	Acceptor splice site-branch site r.2245_2395de; p.? ***^#^***	29	rs1569495747 (13)	Affects splicing	R
3	c.646-1 G>T	Donor splice site	Intron 11	None	Affects splicing	R
4	c.446del	c.446del p.Pro149Leufs^*^6 frameshift deletion (Inframe deletion)	12	rs104886054 (18)	Not available	R
5	c.3700C>T	Nonsense c.3700C>T p.Gln1234^*^	41	Rs281874719 (19)	40^§^	R
6	c.796C>T	Nonsense p. (Arg266^*^)	14	rs104886071 (20)	36^§^	R
7	c. 1117C>T	Nonsense p. (Arg373^*^)	19	rs107829929 (21)	36^§^	R
8	c.1845del	Frameshift p. (Asn616Ilefs^*^2)	25	None	Not available	P
9	c.649_656del	Frameshift p. (Asn217Leufs^*^9)	12	LOVD database	Not available	R
10	c.3274delG	Frameshift p. (Asn1093Thrfs^*^59)	37	None	Not available	P
11	c.2184delG	Frameshift p. (Gly728Glyfs^*^8)	28	LOVD database	Not available	R
12	c.4793_4798delinsTT (*COL4A5*) c.2765 G>T (*COL4A3*)	Frameshift p. (Ser1598Phefs^*^2) (*COL4A5*) Missense p. (Gly922Glu) (*COL4A3*)	49 34	None rs920413118	Not available 26^§^	P
13	c.2015G>A (*COL4A5*) c.5012_5013delGAinsTT (*COL4A3*)	Missense p. (Gly672Asp) (*COL4A5*) stop-lost extension mutation p. (^*^1671Pheext^*^2) (*COL4A3*)	26 52	LOVD database Not available	27^§^ Not available	R P
14	c.2245-40A>G^**^	Acceptor splice site-branch site r.2245_2395de; p.?***^#^***	29	rs1569495747 (13)	Affects splicing	R

*Pathogenicity of Mutations was compared with ARUP database and LOVD database. Presumption of pathogenicity was made by analysis with protein reconstruction software. R = reported as pathogenetic; P = presumed to be pathogenetic*.

* *balanced X inactivation of both wild type and mutant alleles (11)*.

** *skewed X inactivation of wild type allele (11)*.

# *as a consequence of a variant destroying the exon 29 branch point, the sequence from nucleotide r.2245 to r.2395 (exon 29) is deleted from the transcript (11)*.

† *Pathogenicity of intron variants predicted using Alamut software v.2.11 (Interactive Biosoftware, Rouen, France), indicating functional impact of variants with relevant prediction tools included MaxEntScan, NNSPLICE, Human Splicing Finder, SpliceSiteFinder, GeneSplicer predictions tools*.

§ *Pathogenicity of missense variants predicted using the “Combined Annotation Dependent Depletion tool (CADD)”*.

Finally, genetic analysis can be considered complete if it also includes the assessment of the outcome of the male relatives of our patients. Consequently, we evaluated the mean age of ESKD appearance among this population, which resulted similar between two groups (*p* = 0.711) (see Table 3).

**Table 3:** Age of ESKD appearance in male relatives of our patients.

**Pts**	**Male relative affected by XLAS**	**Age of ESKD appearance (years)**
**GROUP 1**		
1	Father	25
2		
3		
4		
5	Grandfather	35
6		
7		
8	Brother	15
9		
10	Father	18
**GROUP 2**		
1		
2	Brother	16
3	Father	30
4		
5		
6	Father	20
7		
8		
9		
10		
11		
12		
13		
14	Uncle	16

### Proteinuria

**Figure 1:**
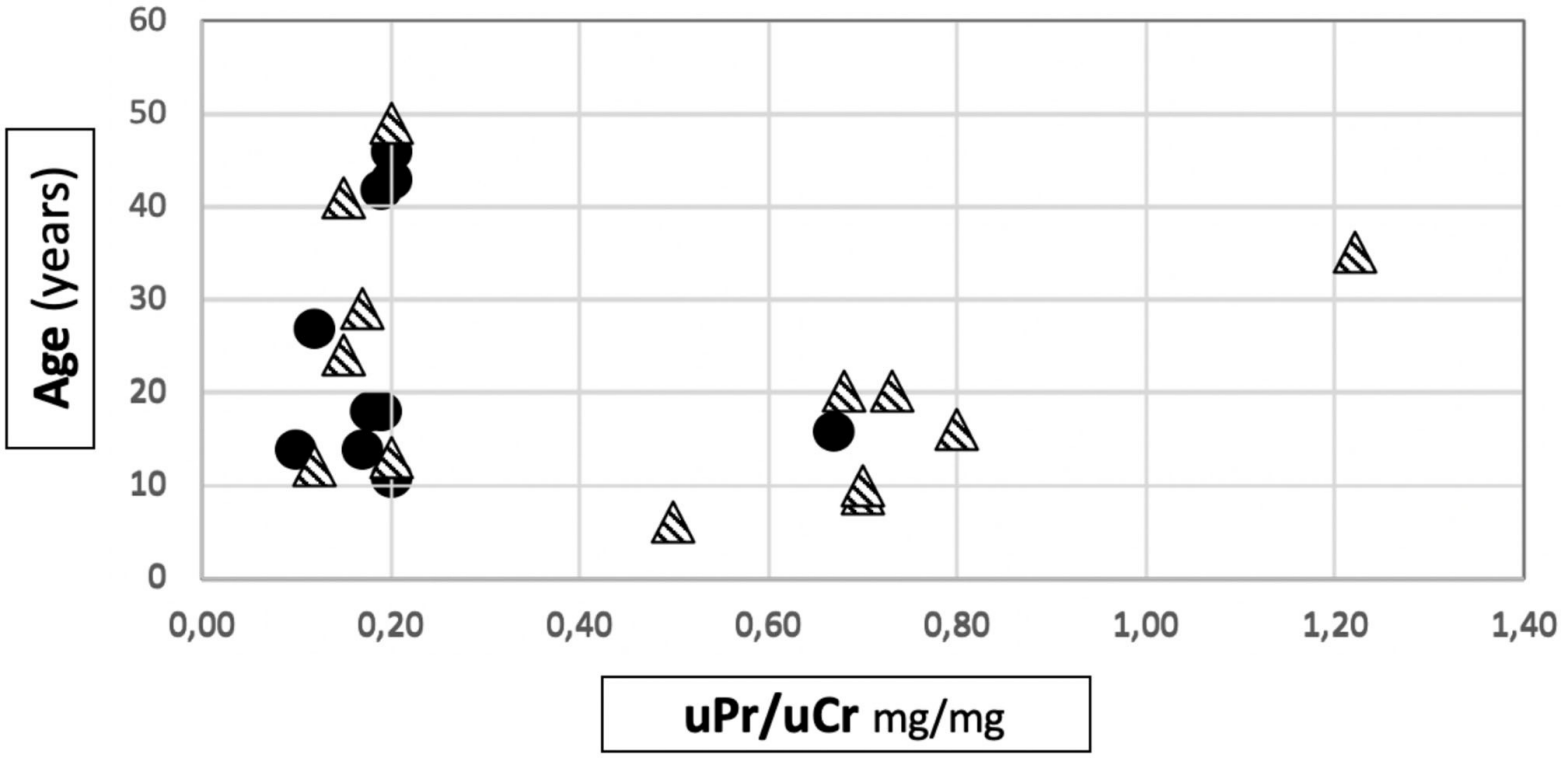
Distribution of age to proteinuria correlation in our cohort. • patients belonging to Group 1. 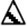 patients belonging to Group 2.

Among patients with Mild variants (Group 1), only one patient out of 10 developed proteinuria, while there were 9 proteinuric cases out of 14 in Group 2 (Fisher's test: *p* = 0.013) (Table 1). Proteinuria occurred at an early age in Group 2 (15.1 ± 9.2 years) (Supplementary Figure 1). In Group 1, the only proteinuric patient developed proteinuria when she was 9 years old. Figure 1 shows the distribution of the age to proteinuria correlation, highlighting the age-related risk for developing proteinuria among the two groups of patients. All proteinuric patients were treated with ACE-I; treatment was started when uPr/uCr was ≥ 0.5 mg/mg. Mean uPr/uCr at last follow up was 0.75 ± 0.18 mg/mg.

### Renal Function

The mean eGFR at the last follow up visit in Group 1 was 119.5 ± 6.2 ml/min/1.73m^2^, while in Group 2 it was 103.4 ± 29.9 ml/min/1.73m^2^. The difference was statistically significant (*p* = 0.027) (Table 1). Anyway, it is interesting to note that an impairment of renal function was only present in 2 patients, both from the severe mutation group: 1 patient developed ESKD when she was 40 years old and 1 had CKD stage 2 (eGFR 75 ml/min/1.73 sm) from the age 34. The former had a digenic form of AS and the latter had a frameshift mutation. The number of patients with CKD/ESKD was not statistically different between two groups (Fisher's test 0.4928, ns), although the mean eGFR was lower in Group 2.

**Figure 2:**
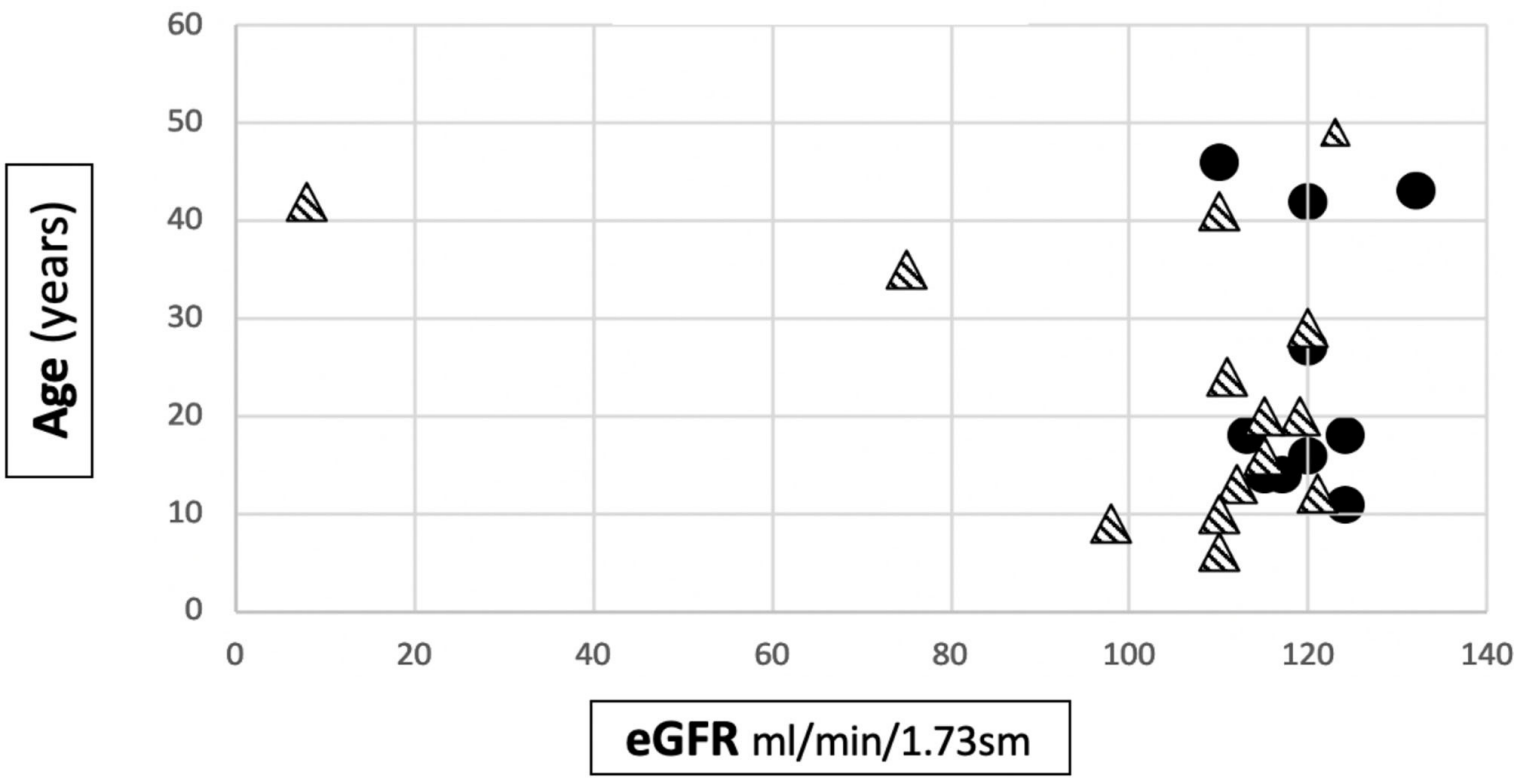
Distribution of age to eGFR correlation in our cohort. • patients belonging to Group 1. 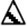 patients belonging to Group 2.

Figure 2 shows the distribution of the age to eGFR correlation in our patients.

All patients had normal blood pressure readings during the entire observation period.

### Extrarenal Involvement

Slight sensorineural auditory deficit was diagnosed in 2 patients belonging to Group 1 (none with ear prosthesis) and in 2 patients from Group 2 (one with an ear prosthesis) (*p* = n.s.). The last patient was prosthetized when she was 18 years old and progressed to ESKD.

No ocular abnormalities were detected in either group, so we did not perform any statistical analyses.

### Histological Frameworks

Renal biopsy was performed in 9/10 pts in Group 1 and in 12/14 in Group 2. In three patients, a renal biopsy was performed in a relative with the same genetic mutation of the index case: a father, a mother and a son.

The analysis of kidney specimens showed a pattern suggestive for AS in all the patients in both groups. Complete ultrastructural features seen at electron microscopy were available for only 6 patients in Group 1 and 10 patients in Group 2 (Table 4). There were no differences in the focal or diffuse presence of these features between the two groups.

**Table 4:** Histological ultrastructural features in our cohort of patients, where available.

**Pts**	**Thinning**	**Thickening**	**Lamellation**	**Basket weave lesions**
**GROUP 1**—**patients with Mild mutations**				
1	+	+	+	–
2	+	+	+	+
3				
4				
5				
6	++	–	+	–
7				
8	++	–	+	–
9	+	–	+	–
10	+	+	+	+
**GROUP 2**—**patients with Severe mutations**				
1	+	–	+	–
2	+	+	+	–
3				
4	+	+	+	–
5				
6	+	+	++	++
7	+	–	+	+
8	++	+	+	+
9	++	+	+	+
10	++	–	+	++
11	+	+	+	+
12				
13				
14	+	+	+	–

*+focal distribution of the feature*.

*++a diffuse distribution of the feature*.

## Discussion

The focus of this study was the evaluation of the genotype-phenotype correlation in 24 females with XLAS. While this correlation is clear in male patients, it remains poorly characterized in females.

In the past, it was thought that females with XLAS had a benign renal course with a normal lifespan and for this reason the disease is still underdiagnosed in women (1). The most extensive study focusing on the natural history of heterozygous females was performed by the European Community Alport Syndrome Concerted Action Study (ECASCAS) and published in 2003 (5). This study reported that hematuria was present from infancy in 95% of 323 girls and women with XLAS, while the prevalence of proteinuria was 75%, and that proteinuria, hypertension and deafness represented risk factors for the progression to nephrotic syndrome and ESKD. The analysis of our cohort confirmed these data, even if proteinuria was found in a lower percentage of patients (41%).

As already defined for a number of chronic glomerular diseases and also confirmed by ECASCAS in 2003 for XLAS (5), the appearance of proteinuria represents a risk factor for progression toward CKD and ESKD. The earlier the appearance of proteinuria, the greater the risk for disease progression (2, 3, 5). Our study shows that the Severe variants expose patients to a greater risk for developing proteinuria and CKD. The average age at onset of proteinuria was 15.1 ± 9.2 years, an early age for females with XLAS (2, 3, 5). Furthermore, in Group 2 there were two patients with CKD/ESKD.

A large study conducted by Bekheirnia et al. involving 175 families in the USA showed a rather specific genotype-phenotype correlation in males (4). In females, the genotype-phenotype correlation was not so clear-cut and the explanation for the wide variability is likely multifactorial. Jais et al. was not able to correlate the type of mutation with the severity of renal or extrarenal disease, or the GBM ultrastructural changes in 195 families (5). They demonstrated the lowest rate of progression to ESKD in patients with Mild mutations, but this finding was not statistically significant. They also detected a large intrafamilial variability of the disease in girls and women and postulated that a possible determinant of this heterogeneity could be skewed XCI (5).

Yakota et al. also reported two females with XLAS in order to evaluate the influence of somatic mosaic variants on developing proteinuria. Both patients had somatic mosaic mutations in *COL4A5* in urinary sediment cells with low variant frequencies (17.9 and 22.1%). One of them only had hematuria, the other had moderate proteinuria despite the fact that the absence of skewed XCI and the presence of a low variant frequency in urinary sediment cells had been hypothesized to be associated with a milder phenotype. In this patient, they also found a heterozigous variant in *COL4A3* and concluded that factors determining severity in female XLAS patients still remain unclear, although digenic inheritance seemingly plays an important role (22).

A recent report published by Yamamura et al. on 275 female XLAS patients showed no correlation between renal-survival curves for patients and the presence or absence of missense mutations (9). However, there are clinical differences between their study and ours: Asian vs. European ethnicity and the lower age at onset of proteinuria in the Yamamura cohort (7 vs. 15.1 yrs). Moreover, the wide age range at diagnosis (0–92 years) in their cohort means that the impact of other aggravating factors, such as hypertension, diabetes, and obesity, on developing proteinuria cannot be ruled out; the lower and more homogenous age at clinical appearance of the disease (10.7 ± 10.4 years) in our case series indicates a minor or null effect of other risk factors.

Based on Savige's classification (8) of Severe and Mild mutations, the appearance of proteinuria was more common in patients with Severe mutations (*p* = 0.013) in our population. Indeed, in Group 2, 9 out of 14 patients with Severe variants developed proteinuria, compared to only 1 in 10 from the group of patients with Mild variants. In addition, a further 2 patients out of 5 without proteinuria in Group 2 had acceptor splice site mutations which, according to Gross, would be expected to have a better prognosis compared to those with severe mutations (3).

Given the small number of patients, we were unable to evaluate the significance of the position of the missense mutations. Gross et al. reported that the first 20 exons influence the progression to ESKD less severely (3), in contrast with the results obtained by Bekheirnia et al., who reported a worse prognosis in patients with the mutation positioned at the 5' end of the gene (4).

To complete the genetic analysis, it could be interesting to speculate on differences in ESKD appearance in male patients with the same mutations. Unfortunately, there is no evidence in the literature regarding the different time of evolution in males with the same specific mutation that we reported in our cohort. However, it is well-known that the same mutation, whether moderate or severe, can give phenotypic variability even within the same sex. Considering our population, Table 3 shows that the age of appearance of ESKD in the male relatives of our patients is not statistically different between the two groups (*p* = 0.711), but the early appearance of ESKD (<35 years of age) confirms that the male phenotype is usually more serious than the female one sharing the same mutation, in which the inactivation of X must always be taken into account (13).

In our case series, one patient was proteinuric and showed a greater XCI of the X-chromosome carrying the wild type allele, and a further two proteinuric patients carried digenic inheritance. One of these patients progressed to ESKD. As regards the skewed XCI ratio, which was evaluated in a single family, a worse renal outcome was seen in the daughter (Patient n°14, Group 2) with a greater inactivation of the wild-type allele (91%) than in her mother (45%), as previously described (13). Severe skewing of XCI in favor of expression of the mutant *COL4A5* has been reported in humans since 1995 (23) and in murine models since 2010 (24). Rehault et al. showed that a modest skewing of X inactivation ratios influences a distinct survival advantage in murine models and that X inactivation ratio correlates with both higher urine protein excretion and plasma urea nitrogen levels at 6 months of age (24). On the other hand, in humans only a few papers have reported a significant correlation between the expression of the X chromosome with a mutated *COL4A5* gene and prognosis (23, 25). Skewing of XCI was tested in different tissues: lymphocytes, cells from renal tissue, urinary cells and the epidermal basement membrane and the results of these papers were often contradictory, even in cases where the same tissues were analyzed (23, 25–29). Therefore, XCI cannot be currently considered as a strong tool for evaluating the renal prognosis of XLAS females, even if it could potentially explain a portion of the observed variability in outcomes.

Another explanation for the wide phenotypic and intrafamilial variability is the presence of digenic inheritance (30). In the past, it was common to discontinue DNA analysis following the identification of one mutation; nowadays, with parallel sequencing, it is possible to evaluate all three type IV collagen genes and identify patients with a two-locus disease. Our patients with digenic mutations seem to confirm this.

Digenic inheritance has also already been reported to have a prognostic role in other kidney diseases, such as congenital nephrotic syndrome (mutations in NPHS1 and NPHS2) (31), autosomal dominant polycystic kidney disease (mutations in PKD1 and PKD2) (32) and Bartter's syndrome (33).

Mencarelli et al. reported on 11 patients with 2 mutations in different COL4 genes, generally more severely affected than the patients with one mutation, who had an intermediate phenotype between the autosomal dominant and the autosomal recessive forms (7). In our cohort, two cases of digenic inheritance (association of a pathogenic mutation of *COL4A5* and *COL4A3*) were present: one had a Severe mutation in COL4A5 and a Mild mutation in COL4A3 and presented with uPr/uCr > 0.5 at 13 years of age, while the second had a Mild mutation in COL4A5 and a Severe variant in COL4A3 and developed ESKD and needed an auricular prosthesis. The latter patient was included in Group 2 because the mutation in COL4A3 gene was labeled as pathogenetic. Consequently, she could not be classified as having a pure missense mutation in COL4A5.

Finally, as hypothesized, eGFR was significantly lower (*p* = 0.027) in patients with Severe variants: in Group 1, eGFR was normal in all patients, while 2 patients in Group 2 had CKD, one of whom progressed to ESKD at the age of 40. Anyway, the number of patients with CKD/ESKD was not statistically different between the two groups. As previously suggested (5), the appearance of proteinuria in XLAS females is a risk factor for CKD, however the time of evolution toward ESKD is actually not calculable.

All patients had a kidney biopsy suggestive of AS, independently of the underlying genetic mutation, suggesting that renal structure and histological features cannot help in arriving at a prognosis. Furthermore, the presence of diffuse or focal features does not appear to correlate with the type of mutation.

A limitation of our study is the number of patients considered. However, this is due to the monocentric design of the study and the rarity of the disease. Despite this, the number of patients could be considered adequate given that it is a single center cohort and the study is prospective in design.

Furthermore, it could be noted that 4 patients, Patient n°3, 4, 5, and 8 in Group 1 were only followed for 2 years. However, we do not believe that this affected our results significantly, because these girls were aged 14, 43, 14, and 18 years old, respectively. So, from a broader perspective, this is the observational time that deserves to be considered in these patients because AS is an inherited disease with clinical signs (hematuria) that appear very early in life. In fact, the mean age at the end of the follow up was very similar in both groups (24.9 ± 13.6 years and 23.2 ± 13.8 years, *p* = 0.659) and this demonstrates that the overall observation time is superimposable between the two groups.

Finally, XCI was only studied in a single family and this precludes any conclusion regarding the effect of wild-type XCI on phenotype severity. Moreover, the aim of this study was not to assess the single effect of XCI on phenotype, which was already described by us in a different paper (13), but to emphasize the role of any non-missense mutations (Severe forms) on the phenotype of female XLAS patients.

In conclusion, in our series of 24 XLAS females, early proteinuria is more frequent in patients with non-missense variants in *COL4A5*. Our data suggest that these patients are also at higher risk for progression toward CKD, even if clear evidence is lacking despite a significant difference in mean eGFR at the end of the follow up. For these reasons, non-missense variants could be considered more severe than missense mutations, as previously hypothesized (5, 8). We also suggest that digenic inheritance and skewed XCI may play a role. Although the severity of female XLAS may be multifactorial, our data support the hypothesis of a genotype-phenotype correlation in this rare population. Consequently, the early use of ACE-I in female XLAS patients with non-missense mutations could be useful, even before the onset of proteinuria (34, 35).

## Data Availability Statement

The datasets presented in this study can be found in online repositories. The names of the repository/repositories and accession number(s) can be found in the article/Supplementary Material.

## Ethics Statement

The studies involving human participants were reviewed and approved by Milano Area 2, in Fondazione IRCCS Ca' Granda Ospedale Maggiore Policlinico, Milan. Written informed consent to participate in this study was provided by the participants' legal guardian/next of kin.

## Author Contributions

AM and MG conceptualized and designed the study, collected clinical data, performed data analysis and interpretation, drafted the article, and approved the final version of the manuscript. EG collected clinical data, performed data analysis, and approved the final version of the manuscript. PC and GS collected clinical data, critically revised the article, and approved the final version of the manuscript. MR, CF, and MB collected clinical data and approved the final version of the manuscript. AR conceptualized and designed the study, critically revised the article, and approved the final version of the manuscript. GM conceptualized and designed the study, performed data interpretation, critically revised the article, and approved the final version of the manuscript. All authors contributed to the article and approved the submitted version.

## Conflict of Interest

The authors declare that the research was conducted in the absence of any commercial or financial relationships that could be construed as a potential conflict of interest.
