# Supplementary material for: X-Linked Alport Syndrome in Women: Genotype and Clinical Course in 24 Cases
Source: Front Med (Lausanne). 2020 Nov 23;7:580376. doi: 10.3389/fmed.2020.580376 (PMC7719790; doi:10.3389/fmed.2020.580376)
Supplement: Supplementary file 1 [file Data_Sheet_1.docx]

**SUPPLEMENTARY MATERIAL**

**GROUP 1**- patients with M variants

| Part 1…  **Patient** |  | **Ethnicity** | **Family history** | **Clinical presentation in family members** | **Age at clinical onset (years)** | **Age at genetic diagnosis (years)** | **Age at first detection of proteinuria (years)** | **uPr/uCr (mg/mg) at onset** | **Peak of uPr/uCr (before RAASi)** | **uPr/uCr al last FUP** | **BP at onset** | **BP at last FUP** |
| --- | --- | --- | --- | --- | --- | --- | --- | --- | --- | --- | --- | --- |
| 1 |  | Caucasian | paternal | HD when he was 16 years old | 2 | 8 | - | - | - | 0,12 | 90/65 | 110/65 |
| 2 |  | Caucasian | no | na | 12 | 16 | - | - | - | 0,18 | 103/55 | 124/85 |
| 3 |  | Caucasian | maternal | ESKD at age of 47 y | 13 | 13 | - | - | - | 0,17 | 107/66 | 113/74 |
| 4 |  | Caucasian | maternal | HD at 60 years of age, SNHL, | 41 | 42 | - | - | - | 0,2 | 105/60 | 110/67 |
| 5 |  | Caucasian | maternal | Only microscopic hematuria | 12 | 14 | - | - | - | 0,10 | 98/57 | 100/64 |
| 6 |  | Caucasian | paternal | SNHL e HD when he was 31 | 23 | 42 | - | - | - | 0,19 | 121/72 | 125/82 |
| 7 |  | Caucasian | mother | Only microscopic hematuria | 6 | 46 | - | - | - | <0,2 | 108/54 | 120/80 |
| 8 |  | Caucasian | maternal | Only microscopic hematuria | 16 | 17 | - | - | - | 0,19 | 120/80 | 123/78 |
| 9 |  | Caucasian | no | na | 1 | 8 | - | - | - | 0,2 | 73/41 | 103/61 |
| 10 |  | Caucasian | paternal | HD from 18 years of age | 3 | 15 | 9 | 0,35 | 0,53 | 0,67 | 95/55 | 115/78 |

| Part 2…  **Patient** | **SNHL** | **Age at SNHL (years)** | **Ocular abnormalities** | **Renal biopsy performed in:** | **Diagnosis at electron microscopy** | **eGFR at onset** | **eGFR at last FUP** | **CKD** | **Age at CKD** | **ESKD** | **Age at ESKD** | **Length of FUP** | **Age at last FUP** |
| --- | --- | --- | --- | --- | --- | --- | --- | --- | --- | --- | --- | --- | --- |
| 1 | no | n.a. | no | Patient | AS | 137 | 120 | no | n.a. | n.a. | n.a. | 25 | 27 |
| 2 | no | n.a. | no | Patient | AS | 139 | 113 | no | n.a. | n.a. | n.a. | 5 | 18 |
| 3 | no | n.a. | no | Patient | AS | 110 | 115 | no | n.a. | n.a. | n.a. | 2 | 14 |
| 4 | Deficit | 40 | no | Patient | AS | 93 | 132 | no | n.a. | n.a. | n.a. | 2 | 43 |
| 5 | no | n.a. | no | Patient | AS | 162 | 117 | no | n.a. | n.a. | n.a. | 2 | 14 |
| 6 | Deficit | 36 | no | Patient | AS | 138 | 120 | no | n.a. | n.a. | n.a. | 19 | 42 |
| 7 | no | n.a. | no | Patient | AS | 143 | 110 | no | n.a. | n.a. | n.a. | 40 | 46 |
| 8 | no | n.a. | no | Patient | AS | 125 | 124 | no | n.a. | n.a. | n.a. | 2 | 18 |
| 9 | no | n.a. | no | Patient | AS | 148 | 124 | no | n.a. | n.a. | n.a. | 10 | 11 |
| 10 | no | n.a. | no | Patient | AS | 142 | 120 | no | n.a. | n.a. | n.a. | 13 | 16 |

**GROUP 2** – patients with S variants

| Part 1…  **Patient** | **Ethnicity** | **Family history** | **Clinical presentation in family members** | **Age at clinical onset (years)** | **Age at genetic diagnosis (years)** | **Age at first detection of proteinuria (years)** | **uPr/uCr (mg/mg) at onset** | **Peak of uPr/uCr (before RAASi)** | **uPr/uCr al last FUP** | **BP at onset** | **BP at last FUP** |
| --- | --- | --- | --- | --- | --- | --- | --- | --- | --- | --- | --- |
| 1 | Caucasian | maternal | Only microscopic hematuria | 2 | 12 | - | - | - | <0.2 | 89/53 | 98/57 |
| 2 | Caucasian | maternal | microscopic hematuria and mild proteinuria at old age | 2 | 45 | - | - | - | <0.2 | 88/56 | 120/70 |
| 3 | Caucasian | paternal | HD at 33 years | 10 | 12 | - | - | - | 0,12 | 103/69 | 103/61 |
| 4 | Caucasian | paternal | HD when he was 27 yo | 9 | 18 | - | 0,1 | - | 0,15 | 99/59 | 116/73 |
| 5 | Caucasian | maternal | n.a. | 4 | 40 | - | - | - | 0,15 | 102/51 | 125/78 |
| 6 | Caucasian | paternal | HD when 20 yo | 7 | 8 | 12 | 0,15 | 0,54 | 0,8 | 105/62 | 114/77 |
| 7 | Caucasian | no | n.a. | 0,7 | 4 | 5 | 0,54 | 0,79 | 0,5 | 88/54 | 125/60 |
| 8 | Caucasian | maternal | Only microscopic hematuria | 27 | 30 | 30 | 0,86 | 1.37 | 1,22 | 120/80 | 120/80 |
| 9 | Chinese | Adopted child | Not available | 4 | 6 | 5 | 1,2 | 1,4 | 0,7 | 115/66 | 107/65 |
| 10 | Caucasian | maternal | Only microscopic hematuria | 1 | 9 | 8 | 0,19 | 0,64 | 0,7 | 95/50 | 110/56 |
| 11 | Caucasian | no | n.a. | 14 | 19 | 14 | 0,57 | 0,69 | 0,68 | 120/60 | 118/72 |
| 12 | Caucasian | mother | Only microscopic hematuria | 24 | 27 | 27 | 0,4 | 0,8 | 0,17 | 113/67 | 105/65 |
| 13 | Caucasian | son | Early proteinyrua | 22 | 40 | 22 | 1,2 | 5,6 | ESKD | 120/60 | 135/90 |
| 14 | Caucasian | maternal | mild proteinuria | 1 | 19 | 13 | 0,12 | 1 | 0,73 | 82/49 | 110/67 |

| Part 2..  **Patient** | **SNHL** | **Age at SNHL (years)** | **Ocular abnormalities** | **Renal biopsy performed in:** | **Diagnosis at electron microscopy** | **eGFR at onset** | **eGFR at last FUP** | **CKD** | **Age at CKD** | **ESKD** | **Age at ESKD** | **Length of FUP** | **Age at last FUP** |
| --- | --- | --- | --- | --- | --- | --- | --- | --- | --- | --- | --- | --- | --- |
| 1 | no | - | no | Patient | AS | 131 | 112 | no | n.a. | n.a. | - | 11 | 13 |
| 2 | no | - | no | Patient | AS | 127 | 123 | no | n.a. | n.a. | - | 47 | 49 |
| 3 | no | - | no | Father | AS | 180 | 121 | no | n.a. | n.a. | - | 2 | 12 |
| 4 | no | - | no | Patient | AS | 146 | 111 | no | n.a. | n.a. | - | 15 | 24 |
| 5 | no | - | no | Mother | AS | 156 | 110 | no | n.a. | n.a. | - | 37 | 41 |
| 6 | no | - | no | Patient | AS | 161 | 115 | no | n.a. | n.a. | - | 9 | 16 |
| 7 | no | - | no | Patient | AS | 177 | 110 | no | n.a. | n.a. | - | 5,3 | 6 |
| 8 | no | - | no | Patient | AS | 91 | 75 | yes | 34 | n.a. | - | 8 | 35 |
| 9 | Deficit | 8 | no | Patient | AS | 122 | 98 | no | n.a. | n.a. | - | 5 | 9 |
| 10 | no | - | no | Patient | AS | 139 | 110 | no | n.a. | n.a. | - | 9 | 10 |
| 11 | no | - | no | Patient | AS | 140 | 119 | no | n.a. | n.a. | - | 6 | 20 |
| 12 | no | - | no | Patient | AS | 133 | 120 | no | n.a. | n.a. | - | 5 | 29 |
| 13 | Prosthesis | 18 | no | Patient | AS | 73.7 | 8 | yes | 22 | yes | 40 | 20 | 42 |
| 14 | no | - | no | Patient | AS | 123 | 115 | no | n.a. | no | - | 17 | 20 |

**Table S1:** Clinical, laboratory and biopsy findings in our patients. eGFR is expressed as ml/min/ 1.73sm

**
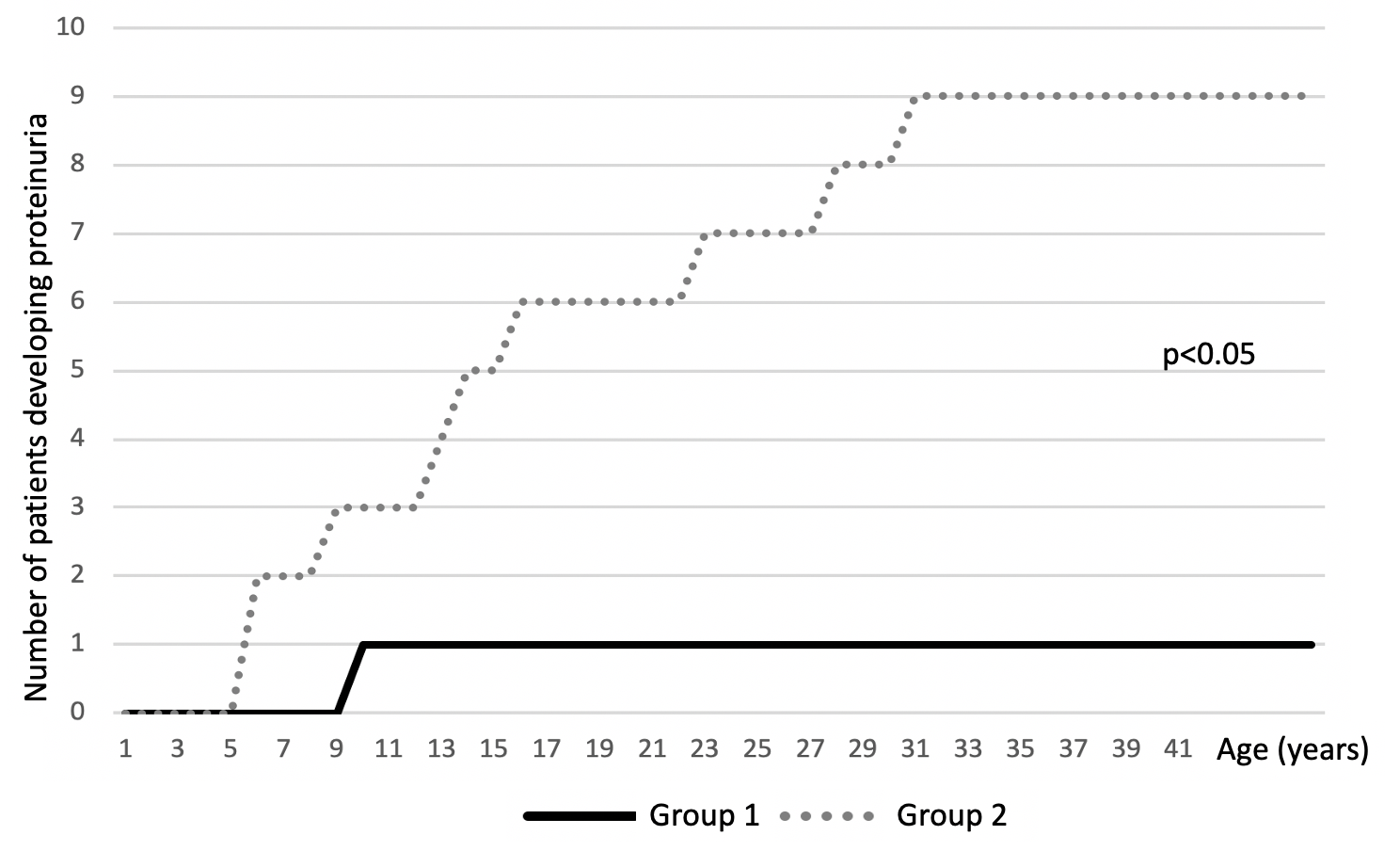
**

**Figure S1.** Time to appearance of proteinuria in Groups 1 and 2
